# Effects of exogenous proteins on retrogradation properties of rice starch, gel properties of raw rice flour and eating quality of fresh wet rice noodles

**DOI:** 10.3389/fnut.2026.1915300

**Published:** 2026-09-03

**Authors:** Shanni Wang, Yike Zhang, Lei Wang, Jinshui Wang, Yaochuang Hu, Fan Wu, Hanyue Kang, Hongwei Wang, Zuman Dou

**Affiliations:** 1 College of Biological Engineering, Henan University of Technology, Zhengzhou, China; 2 Future Cereal Center of Henan University of Technology & Arawana, College of Food Science and Engineering, Henan University of Technology, Zhengzhou, China; 3 College of International Education, Henan University of Technology, Zhengzhou, China; 4 College of Food and Biological Engineering, Zhengzhou University of Light Industry, Zhengzhou, Henan, China; 5 College of Ocean Food and Biological Engineering, Jimei University, Xiamen, China; 6 Enshi Tujia and Miao Autonomous Prefecture Academy of Agricultural Sciences, Enshi, China

**Keywords:** exogenous proteins, fresh wet rice noodles, gelatinization, low-field nuclear magnetic resonance, small-angle X-ray scattering, texture analysis

## Abstract

**Introduction:**

Starch retrogradation severely deteriorates texture and shelf life of fresh wet rice noodles, while the distinct regulatory mechanisms of wheat gluten (WG) and soy protein isolate (SPI) on rice starch gel structure and noodle quality remain insufficiently clarified.

**Methods:**

Indica rice flour was supplemented with 0–6% WG or SPI. Multi-scale characterizations including pasting, thermal, gel texture, water distribution, microstructure and starch lamellar structure and noodle cooking qualities were performed to compare their anti-retrogradation effects and fresh wet rice noodle quality characteristics.

**Results:**

Both WG and SPI inhibited gelatinization and retrogradation of rice starch, restricted water migration, restrained gel structural rearrangement and slowed the growth of gel hardness during storage. However, WG and SPI regulated noodle quality through different pathways. WG optimized noodle texture by consolidating gel networks, whereas SPI inhibited starch retrogradation and water movement to slow noodle hardening. SPI performed better in alleviating rice noodle stiffness, and samples containing 6% SPI presented a hardness increase rate as low as 1.44 g·d^−1^ after 14 days of storage. Within the WG addition range of 1–6%, increasing WG dosage elevated noodle hardness while reducing adhesiveness and cooking loss. Supplementation of SPI over 3% negatively affected noodle quality, which could be attributed to its strong inhibition against starch retrogradation.

**Discussion:**

This study systematically distinguishes the differential regulation modes of WG and SPI on multi-scale starch structural rearrangement and fresh wet rice noodle quality, offering theoretical support and technical reference for delaying rice noodle retrogradation and extending shelf life via exogenous protein fortification.

## 1 Introduction

Protein is the second most abundant component in rice, and is distributed both on the surface and within starch granules, where it forms a spatial network structure with starch and consequently influences the gelatinization and retrogradation behaviors of rice-based products (1). Based on solubility characteristics, rice proteins are classified into four categories: prolamins, globulins, albumins, and glutelins. Previous studies have demonstrated that rice proteins affect the properties of rice noodles through two primary mechanisms (2). First, variations in protein content alter water-binding capacity during cooking. Second, protein–protein and protein–starch interactions, including disulfide bonds, covalent linkages, and non-covalent associations, influence the structural and physicochemical properties of the starch matrix. Baxer et al. (3) reported that glutelin restricts the penetration of water into starch granules during gelatinization. Fan et al. (4) further demonstrated that proteins inhibit rice starch retrogradation and hinder the formation of ordered structures through competitive hydration and non-covalent interactions.

Starch retrogradation is the process by which gelatinized starch gradually transforms from a disordered amorphous into a more ordered and crystalline structure during cooling and storage (5). This process can be divided into short-term retrogradation and long-term retrogradation. Short-term retrogradation is primarily attributed to the rapid reassociation of amylose molecules and serves as the basis for gel network formation, whereas long-term retrogradation involves the gradual recrystallization of amylopectin throughout storage (6). Progressive starch retrogradation has detrimental effects on rice noodle quality. The increasing compactness of the gel network results in higher hardness and reduced palatability (7). Simultaneously, structural contraction promotes dehydration, resulting in deterioration of textural properties and a shortened shelf life. Consequently, starch retrogradation remains a major challenge limiting the industrial production and storage stability of rice noodles. In recent years, the incorporation of exogenous proteins has attracted considerable attention as an effective strategy for regulating starch retrogradation and improving gel network characteristics. WG, a natural protein extracted from wheat, with a protein content exceeding 75%, consists primarily of glutenin and gliadin. Upon hydration, WG forms a viscoelastic three-dimensional network that can restrict starch chain mobility and rearrangement through physical entrapment (8). SPI, a high-quality purified plant protein extracted from soybeans, possesses excellent emulsifying and gel-forming properties. The numerous polar groups on SPI molecules can compete with starch for water and interfere with the ordered aggregation of starch molecules, hydrogen bonding, and other molecular interactions (9). Wang et al. (10) reported that increasing protein content resulted in incomplete starch gelatinization and inhibited long-term retrogradation by reducing water migration within the gel matrix. Similarly, Xu et al. (11) demonstrated that SPI significantly improved the water-holding capacity of fresh wet rice noodles and retarded hardening during storage by slowing the retrogradation process. Existing studies have separately verified that both WG and SPI can effectively interfere with starch retrogradation. Nevertheless, the two proteins exhibit distinct molecular characteristics and network formation patterns. Accordingly, it is necessary to simultaneously evaluate their comprehensive effects at different supplementation levels and clarify the differential mechanisms by which they regulate the quality of fresh wet rice noodles from a multi-scale structural perspective.

This study systematically investigates the differential regulation of rice flour gel properties and eating quality of fresh wet rice noodles by wheat gluten and soy protein isolate at various supplementation levels, and analyzes the functional differences of the two exogenous proteins modulating noodle quality from a multi-scale structural perspective. Gradient addition levels ranging from 0 to 6% were adopted to compare the differences of the two proteins on pasting behaviors, thermal properties, water distribution, micromorphology, starch lamellar molecular structure and cooking texture of rice noodles. Employing multiple multi-scale characterization techniques, including low-field nuclear magnetic resonance, confocal laser scanning microscopy, and small-angle X-ray scattering, this study elucidates the relationships between protein addition level, the multi-scale structural evolution of starch, and the eating and storage qualities of fresh wet rice noodles. The findings provide a theoretical basis for extending the shelf life and improving the quality of rice noodles through protein fortification and contribute to a deeper understanding of the role of protein composition and structure in regulating starch retrogradation.

## 2 Materials and instruments

### 2.1 Materials

Indica rice (moisture: 12.99%, total starch: 73.80%, amylose content: 19.42%, protein content: 7.33%) was purchased from Yihai Kerry Grain, Oil & Food Co., Ltd. (Nanchang, Jiangxi Province, China). Wheat gluten (WG; moisture: 9.53%, protein content: 82.36%, fat content: 2.11%, ash content: 2.32%) was obtained from Yihai Kerry Starch Technology Co., Ltd. (Dongguan, Guangdong Province, China). Soy protein isolate (SPI; moisture: 6.68%, protein content: 90.03%, fat content: 0.80%, carbohydrate content: 2.05%) was supplied by Linyi Shansong Biological Products Co., Ltd. (Linyi, Shandong Province, China). Analytical-grade potassium bromide was obtained from PIKE Technologies (Madison, Wisconsin, USA).

### 2.2 Sample preparation

Rice grains were soaked in water for 4 h, milled, and passed through a 100-mesh sieve. The resulting rice flour was dried at 40 °C and subsequently blended with WG or SPI at concentrations of 0, 1, 3, and 6% (w/w, calculated based on total rice flour weight), as determined through systematic preliminary experiments. The resulting samples were designated as Control, WG-1, WG-3, WG-6, SPI-1, SPI-3, and SPI-6. For gel preparation, each flour mixture was dispersed in water and heated at 95 °C for 30 min to achieve complete gelatinization. The gelatinized samples were then stored at 4 °C for predetermined periods to obtain gel samples for texture profile analysis and stress relaxation measurements.

For noodle preparation, the blended flour was mixed with water to form a rice slurry. The slurry was gelatinized in a rice noodle machine (SZ-30 rice noodles machine, Xuzhong Food Machinery Co., Ltd., Guangzhou) at 90 °C and extruded through a die with a diameter of 2 mm to produce fresh wet rice noodles.

One portion of the fresh wet rice noodles was directly stored under constant conditions of 35 °C and 85% relative humidity for 6 h for immediate quality evaluation, while the other portion was sealed in polyethylene bags and stored in a 4 °C refrigerator for 7 or 14 days for retrogradation treatment. All samples were subjected to further analyses, including moisture distribution, lamellar structure, microstructure, and eating quality evaluation.

### 2.3 Determination of pasting properties

According to the method proposed by Wu et al. (12) With slight modifications, the pasting properties of rice flour were determined using a Rapid Visco Analyzer (RVA; Perten Instruments, Sweden). Rice flour (3.0 g, adjusted to a 12% moisture basis) was dispersed in 25 mL of distilled water in an RVA canister. The measurement program consisted of equilibration at 50 °C for 1 min, heating to 95 °C at a stirring speed of 960 rpm, holding at 95 °C for 2.5 min, and cooling back to 50 °C at a stirring speed of 160 rpm.

### 2.4 Determination of thermodynamic properties

The thermodynamic properties of samples were analyzed using a differential scanning calorimeter (DSC250, TA Instruments, New Castle, DE, USA) according to the method of Xiong et al. (13) with appropriate modifications. Approximately 3 mg of the sample was weighed into an aluminum DSC pan and mixed with 6 μL of distilled water. The pan was hermetically sealed and equilibrated at room temperature for 24 h prior to analysis. An empty sealed pan was used as the reference. Samples were scanned from 20 °C to 120 °C at a heating rate of 10 °C/min.

### 2.5 Determination of gel hardness

The gel samples were prepared by dispersing 12 g of each flour blend in distilled water to obtain a rice slurry with a solids content of 15% (w/w). The slurry was magnetically stirred for 8 min and then gelatinized in a water bath at 95 °C for 30 min. After cooling, the samples were stored at 4 °C for 1 d, 7 d, 14 d, and 21 d to allow retrogradation. Texture profile analysis (TPA) was performed using a texture analyzer (PHS-25 TA-XT Plus, Stable Micro Systems, Surrey, UK) equipped with a P/6 cylindrical probe. The testing conditions were as follows: pre-test speed, 1 mm/s; test speed, 1 mm/s; post-test speed, 2 mm/s; compression distance, 10 mm; and trigger force, 5 g. The hardness change rate (ΔHardness) was calculated according to Equation 1:


$$\Delta \text{Hardness}/g\cdot d^{-1}=\frac{H_{n}-H_{0}}{n}$$ (1)

where H_n_ is the hardness of the gel after n days of storage (g), H_0_ represents the hardness on day 0 (g), and n denotes the storage time (d).

### 2.6 Determination of gel stress relaxation

The stress relaxation properties of the gel samples were determined according to the method of Jie et al. (14) with minor modifications. Briefly, 24 g each of flour blend was dispersed in distilled water to obtain a slurry with a solids content of 25% (w/w). The slurry was magnetically stirred for 8 min and gelatinized in a water bath at 95 °C for 30 min. The resulting gel was transferred into a cylindrical mold and stored at 4 °C for 12 h prior to analysis. A stress relaxation test was carried out on a cylindrical gel block with a diameter of 25 mm and a height of 15 mm using a P/35 probe. The test conditions are as follows: pre-test speed, 2 mm/s; test speed, 1 mm/s; post-test speed, 2 mm/s; compression strain, 30%; and relaxation time, 180 s. A three-element Maxwell model was used to fit the stress relaxation curves. The model equations are shown in Equations 2, 3:


$$\sigma =\varepsilon_{0}(E0+E_{1}e^{-\frac{t}{\tau }})$$ (2)

$$\tau =\frac{\eta }{E_{1}}$$ (3)

Where *σ* is the stress (or force) exerted on the sample at time t (N); *ε*_0_ is the initial deformation (%); *E*_0_ is the equilibrium elastic modulus (MPa); *E*_1_ is the decay elastic modulus (MPa); *t* is the relaxation tims (s); *τ* is the relaxation constant (s); and *η* is the viscosity coefficient (MPa·s).

### 2.7 Low-field nuclear magnetic resonance

The modified distribution was analyzed according to the method of Zhang et al. (15). Accurately weigh 0.8 g of gel samples aged at 4 °C for 1 d and 7 d, then place them into NMR tubes for measurement. The operating parameters were as follows: sampling frequency (SW), 200 kHz; sampling interval (TW), 3,000 ms; and number of scans (NS), 16.

### 2.8 Fourier transform infrared (FTIR) spectroscopy

Gel samples were freeze-dried, ground, and passed through a 100-mesh sieve. The resulting powders were mixed with KBr at a mass ratio of 1:100 and compressed into transparent pellets. FTIR spectra were recorded using a Fourier transform infrared spectrometer (IRAffinity-1S, Shimadzu, Kyoto, Japan) over the wavenumber range of 400–4,000 cm^−1^, with a spectral resolution of 4 cm^−1^ and 32 scans per sample. Fourier self-deconvolution was performed using OMNIC software to quantify the relative intensities of characteristic absorption bands and evaluate changes in molecular structure.

### 2.9 Confocal laser scanning microscopy (CLSM)

According to the method of Dai et al. (16), part of the gel samples was stored at a − 18 °C freezer for freezing, while the other part was aged in a 4 °C refrigerator for 7 days and then transferred to the −18 °C freezer for freezing. The samples were fixed onto the sample stage, and sections with a thickness of 20 μm were prepared using a cryostat microtome. The sections were placed on glass slides and stained for 20 min with a mixed staining solution consisting of 0.25% (w/v) fluorescein isothiocyanate and 0.025% (w/v) rhodamine B at a volume ratio of 1:1. After staining, the sections were rinsed with distilled water and observed using a confocal laser scanning microscope (FV3000, Olympus, Japan). Excitation wavelengths of 488 nm and 568 nm were used for FITC and rhodamine B, respectively. ImageJ software was employed to quantify the protein area based on the acquired CLSM images. The image acquisition and quantitative analysis parameters were kept consistent for all samples.

### 2.10 Determination of the supramolecular structure

According to the method of Zhang et al. (17) with minor modifications, the changes in the lamellar structure of the samples were determined by small-angle X-ray scattering (SAXS) (Xeuss 2.0, Xenocs, Franch). Rice noodle samples were freeze-dried, ground, and passed through a 100-mesh sieve. The resulting powder was dispersed in distilled water at a ratio of 1: 4 (w/v) and equilibrated overnight. The starch suspension was then transferred into the sample holder for SAXS measurements to obtain one-dimensional scattering profiles. The SAXS measurements were performed at a wavelength of 1.54189 Å with an exposure time of 5 min.

The fractal structure parameters were calculated according to Equation 4:


$$I(q)∼q^{\alpha }$$ (4)

where q is the scattering vector (nm), and α is the slope obtained from linear fitting in the low-*q* region of the double-logarithmic SAXS plot.

The semi-crystalline lamellar thickness *d*_Bragg_ was calculated according to the Woolf-Bragg equation, as shown in Equation 5:


$$d_{\text{Bragg}}=\frac{2\pi }{q}$$ (5)

The one-dimensional correlation function was used to characterize the lamellar structure of starch according to Equation 6:


$$L(r)=\frac{\int_{0}^{\infty }I(q)q^{2}\cos(qr)\mathrm{dq}}{\int_{0}^{\infty }I(q)q^{2}\mathrm{dq}}$$ (6)

where r is the real-space distance, I(q) is the scattering intensity, *q* is the scattering vector, and d corresponds to the second maximum of L(r) representing the repeat distance of the starch lamellar structure.

### 2.11 Quality determination of rice noodles

#### 2.11.1 Color measurement

After preheating the color difference meter, arrange the rice noodles in parallel without gaps, and record the values of L* (lightness), a* (red-green value), and b* (yellow-blue value). The whiteness index W was calculated according to Equation 7:


$$W=100-\sqrt{{\left(100-L^{*}\right)}^{2}+{\left(a^{*}\right)}^{2}+{\left(b^{*}\right)}^{2}}$$ (7)

#### 2.11.2 Cooking properties

Rice noodles (10 g) were cooked in 200 mL of boiling water for 2 min. The cooking water was collected and diluted to 250 mL in a volumetric flask. Subsequently, 50 mL of the diluted cooking water was transferred to a pre-weighed aluminum dish and dried at 105 ± 2 °C to a constant weight. Cooking loss was calculated according to Equation 8:


$$\text{Cooking Loss}/\%=\frac{5M1}{M_{0}(1-W)}$$ (8)

Where: *M*_0_ is the mass of rice noodle sample (g), *M*_1_ is the mass of solids recovered from the cooking water after drying (g), and *W* is the moisture content of the rice noodles (%).

#### 2.11.3 Textural properties of cooked rice noodles

The adhesiveness of cooked rice noodles was determined using a texture analyzer equipped with an HDP/PFS probe. The testing parameters were as follows: pre-test speed, 1 mm/s; test speed, 0.8 mm/s; post-test speed, 10 mm/s; deformation level, 70%; and trigger force, 10 g.

Hardness measurements using an A/LKB-F probe and expressed as the maximum peak force (g). The testing conditions were as follows: pre-test speed, 1 mm/s; test speed, 0.17 mm/s; post-test speed, 10 mm/s; and compression distance, 4.7 mm.

Tensile properties were determined using an A/SPR probe and expressed as the breaking force (g). The measurement parameters included an initial probe separation of 10 mm, pre-test speed of 2 mm/s, test speed of 2 mm/s, and post-test speed of 8 mm/s.

### 2.12 Statistical analysis

All experiments were performed in triplicate. Statistical analyses were conducted using SPSS 22.0 software. Significant differences among means were evaluated using Duncan’s multiple range test at a significance level of *p* < 0.05. The results are expressed as mean ± standard deviation. Graphs were generated using Origin 2021 software.

## 3 Results and discussion

### 3.1 Pasting properties

**Figure 1:**
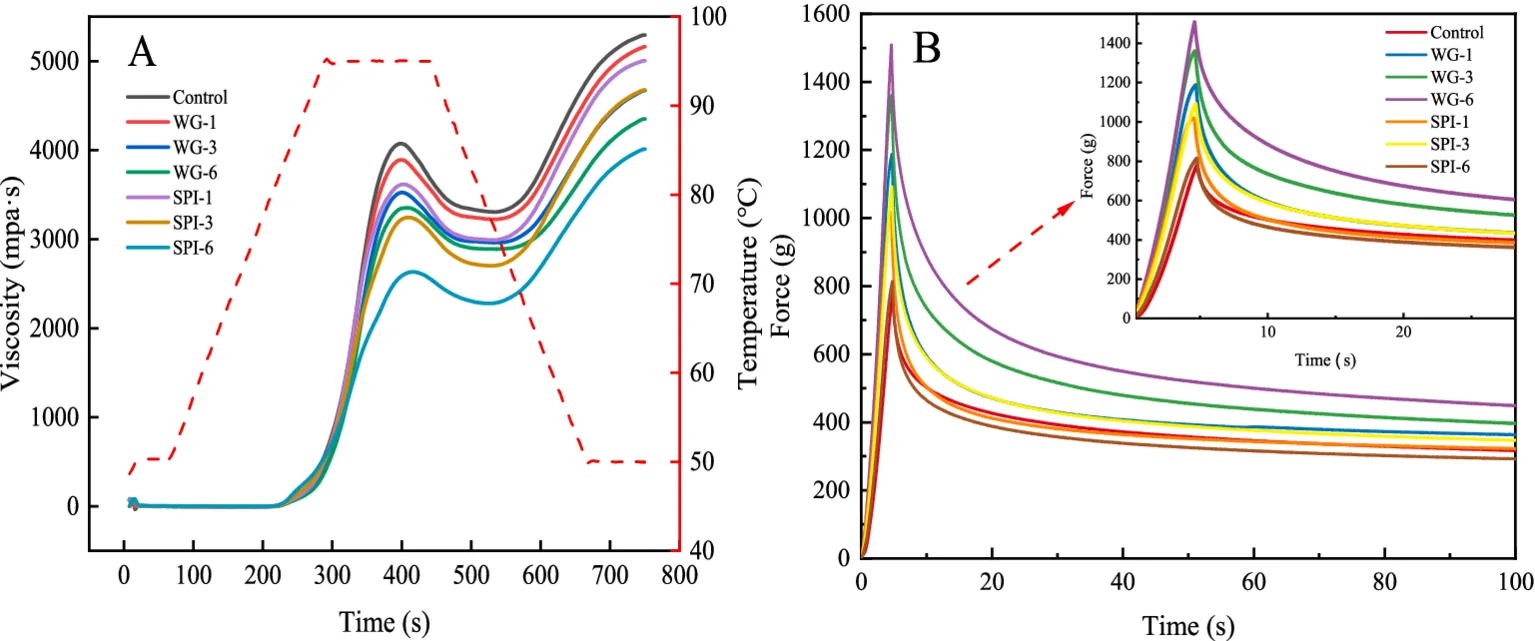
Pasting property curves **(A)** and stress relaxation curves **(B)** of rice flour blended with exogenous proteins. Control: pure rice flour; WG-1, WG-3 and WG-6: samples with 1, 3 and 6% wheat gluten addition, respectively; SPI-1, SPI-3 and SPI-6: samples with 1, 3 and 6% soy protein isolate addition, respectively.

The effects of WG and SPI on the pasting properties of rice noodles are presented in Figure 1 and Table 1. As illustrated in Figure 1A, the viscosity parameters decreased significantly with increasing levels of both WG and SPI, with SPI exerting a markedly greater effect than WG. This reduction can be attributed to the adsorption of protein molecules onto the surface of starch granules, forming a steric barrier that restricts water penetration into the crystalline regions of starch and consequently suppresses starch hydration and swelling. Furthermore, the hydrophilic and hydrophobic groups of SPI exhibit stronger interactions with starch molecules, resulting in a more pronounced inhibition of starch granule swelling and viscosity development (18). As shown in Table 1, the breakdown viscosity decreased progressively with increasing WG and SPI contents, and the SPI-6 sample exhibited the lowest value of 351 mPa·s among all treatments. This result suggests that the presence of proteins enhanced the thermal and shear stability of starch granules during gelatinization. Protein molecules adsorbed onto the granule surface may form a protective layer that reduces granule disruption under high-temperature shear conditions, thereby improving resistance to mechanical breakdown (19).

**Table 1:** Pasting properties of rice flour with exogenous protein addition.

Sample	Peak viscosity/mPa·s	Trough viscosity/mPa·s	Breakdown value/mPa·s	Final viscosity/mPa·s	Setback Value/mPa·s	Pasting temperature/°C
Control	3,860 ± 6^a^	3,175 ± 11^a^	685 ± 17^a^	5,241 ± 33^a^	2067 ± 43^a^	89.40 ± 0.14^f^
WG-1	3,790 ± 5^b^	3,159 ± 21^a^	631 ± 25^b^	5,076 ± 21^b^	1918 ± 42^b^	90.20 ± 0.14^e^
WG-3	3,566 ± 15^d^	2,972 ± 13^c^	594 ± 1^bc^	4,660 ± 15^c^	1,688 ± 28^c^	90.95 ± 0.07^c^
WG-6	3,366 ± 18^e^	2,822 ± 11^d^	545 ± 29^d^	4,349 ± 4^e^	1,528 ± 6^d^	91.78 ± 0.04^a^
SPI-1	3,677 ± 14^c^	3,070 ± 7^b^	607 ± 7^bc^	5,033 ± 41^b^	1963 ± 48^b^	90.18 ± 0.04^e^
SPI-3	3,235 ± 16^f^	2,646 ± 23^e^	589 ± 7^c^	4,558 ± 27^d^	1913 ± 4^b^	90.55 ± 0.07^d^
SPI-6	2,599 ± 47^g^	2,248 ± 44^f^	351 ± 3^e^	3,973 ± 57^f^	1725 ± 13^c^	91.43 ± 0.11^b^

Values followed by the different letter in the same column are significantly different at the 5% level.

The setback viscosity of the WG group was lower than that of the SPI group at each corresponding addition level, demonstrating that WG exerted a stronger inhibitory effect on short-term starch retrogradation during cooling. This can be explained by the fact that WG forms a continuous network which restrains amylose migration more efficiently, thus resulting in a remarkably lower short-term retrogradation value relative to SPI (16). Gelatinization temperature represents the minimum temperature at which starch granules begin to hydrate and swell. The gelatinization temperature of each WG and SPI addition group showed an upward trend with the increase in the addition amount. This can be attributed to competition between proteins and starch for available water, which limits starch hydration. Furthermore, protein-starch interactions, particularly hydrogen bonding, may stabilize the crystalline regions of starch granules and increase the energy required for their disruption, thereby delaying gelatinization and elevating the pasting temperature (20).

### 3.2 Thermal properties

The thermal properties of rice-flour blends are presented in Table 2. The addition of both WG and SPI increased the onset temperature *T*_0_, peak temperature *T*_P,_ and conclusion temperature *T*_C_ of the ungelatinized rice flour samples compared with the control and showed a continuous upward trend with increasing protein content. This result suggests that exogenous proteins interact with starch molecules and strengthen intermolecular associations within the system, thereby enhancing the thermal stability of starch granules and increasing the energy required for gelatinization. Similar observations have been reported by Xu et al. (11). Among all samples, the ungelatinized rice flour exhibited the highest gelatinization enthalpy Δ*H* of 12.77 J·g^−1^, indicating the presence of a highly ordered native crystalline structure (21). Following gelatinization, the Δ*H* values of samples retrograded for 1 d decreased markedly, reflecting the extensive disruption of the native starch crystalline regions during heating and the limited formation of newly ordered structures during the initial stage of retrogradation. As the storage period was extended to 7 d, Δ*H* increased significantly, indicating that starch chains gradually reassociated and formed new hydrogen-bonded structures, resulting in the development of more ordered retrograded starch crystallites (22). The incorporation of WG and SPI reduced the Δ*H* values of the retrograded samples, and the reduction became more pronounced as protein content increased (12). One possible explanation is that proteins compete with starch for available water and interfere with starch chain mobility, thereby suppressing molecular reassociation and crystallization during retrogradation (23). Comparison of the 1-d and 7-d retrogradation data further revealed that SPI exerted a stronger inhibitory effect on starch retrogradation than WG. The lower ΔH values observed in SPI-containing samples indicate that SPI more effectively delays the rearrangement and ordering of starch molecular chains, thereby suppressing the development of retrograded crystalline structures during storage.

**Table 2:** Thermodynamic characteristic parameters of rice flour with exogenous protein addition.

Sample	Ungelatinized raw flour			Retrogradation 0 d	Retrogradation 1 d	Retrogradation 7 d
	*T*_0_/°C	*T*_P_/°C	*T*_C_/°C	Δ*H*/J·g-1	Δ*H*/J·g-1	Δ*H*/J·g-1
Control	74.28 ± 0.03d	78.19 ± 0.13b	84.06 ± 0.04de	12.77 ± 0.11a	6.35 ± 0.17a	10.01 ± 0.18a
WG-1	74.31 ± 0.08 cd	78.28 ± 0.06b	84.29 ± 0.06bc	12.23 ± 0.25ab	5.49 ± 0.34b	9.94 ± 0.30a
WG-3	74.40 ± 0.03bc	78.34 ± 0.01b	84.59 ± 0.13a	10.43 ± 0.10c	5.26 ± 0.11b	8.26 ± 0.20b
WG-6	74.49 ± 0.04b	78.35 ± 0.01b	84.64 ± 0.06a	10.26 ± 0.16c	3.63 ± 0.21c	6.96 ± 0.18c
SPI-1	74.32 ± 0.01 cd	78.19 ± 0.04b	84.01 ± 0.07e	12.35 ± 0.20a	5.43 ± 0.16b	8.03 ± 0.11b
SPI-3	74.44 ± 0.06b	78.42 ± 0.17b	84.22 ± 0.06 cd	11.70 ± 0.33b	4.98 ± 0.23b	5.20 ± 0.17d
SPI-6	74.93 ± 0.04a	78.82 ± 0.31a	84.41 ± 0.07b	10.43 ± 0.47c	3.08 ± 0.17d	4.12 ± 0.24e

Values followed by the different letter in the same column are significantly different at the 5% level.

### 3.3 Gel hardness properties

The effects of exogenous protein addition on the hardness of rice noodle gels during storage are presented in Table 3. The hardness of all samples increased continuously with storage time. This increase can be attributed to the progressive retrogradation of starch following gelatinization, during which starch chains gradually reassociate and form more ordered crystalline structures. The resulting densification of the gel network leads to an increase in gel hardness. The control group exhibited the greatest increase in hardness, reaching 189.27 g after 21 days of storage. In contrast, the hardness values of the WG-6 and SPI-6 groups were 126.97 g and 83.52 g, respectively, and the ΔHardness of the SPI-6 group sample was only 2.9 g·d^−1^. These results demonstrate that both WG and SPI effectively retarded gel hardening during storage, and that the inhibitory effect became more pronounced as protein content increased (4). At equivalent addition levels, SPI exhibited a significantly stronger inhibitory effect on hardness development than WG. This finding is consistent with the DSC results, which showed that SPI more effectively reduced the Δ*H* of starch during storage. The superior anti-retrogradation effect of SPI may be attributed to its abundant hydrophilic and hydrophobic functional groups, which facilitate interactions with starch molecules and hinder starch chain reassociation through steric effects and competitive hydration (24). In addition, this may be attributed to the higher protein content of SPI (90.03%) compared with WG (82.36%), which exerts a more prominent dilution effect on the starch concentration within the rice flour system and consequently reduces the total starch content in the whole system effectively. Consequently, SPI more effectively suppresses the formation of ordered retrograded structures and delays gel hardening.

**Table 3:** Hardness changes of rice flour gels at different aging times.

Sample	Hardness/g					ΔHardness/g·d^−1^			
	0 d	1 d	7 d	14 d	21 d	1 d	7 d	14 d	21 d
Control	35.47 ± 1.45^d^	46.00 ± 1.05^d^	101.83 ± 2.49^a^	153.58 ± 1.90^a^	189.27 ± 7.81^a^	10.53 ± 0.40^a^	9.48 ± 0.57^a^	8.44 ± 0.04^a^	7.33 ± 0.30^a^
WG-1	41.48 ± 1.25^c^	50.45 ± 0.74^c^	93.25 ± 3.55^b^	125.80 ± 6.74^b^	157.15 ± 0.81^b^	8.97 ± 0.51^b^	7.40 ± 0.33^b^	6.03 ± 0.39^b^	5.51 ± 0.02^b^
WG-3	50.14 ± 0.30^b^	57.59 ± 0.26^b^	87.59 ± 0.50^c^	110.70 ± 0.40^c^	134.96 ± 0.71^c^	7.45 ± 0.04^cd^	5.35 ± 0.03^c^	4.33 ± 0.05^c^	4.04 ± 0.05^c^
WG-6	53.76 ± 0.80^a^	59.97 ± 0.47^a^	84.07 ± 1.02^c^	100.92 ± 6.79^d^	126.97 ± 2.10^d^	6.22 ± 0.33^d^	4.33 ± 0.25^de^	3.37 ± 0.54^d^	3.49 ± 0.13^d^
SPI-1	29.96 ± 2.35^e^	38.93 ± 1.14^e^	65.53 ± 0.61^d^	81.75 ± 0.37^e^	93.82 ± 1.05^e^	8.98 ± 1.21^b^	5.08 ± 0.42^cd^	3.70 ± 0.20^cd^	3.05 ± 0.06^e^
SPI-3	29.99 ± 0.30^e^	38.54 ± 0.06^e^	64.68 ± 0.95^d^	79.03 ± 1.05^ef^	89.10 ± 1.11^ef^	8.55 ± 0.24^bc^	4.96 ± 0.09^cd^	3.50 ± 0.10^d^	2.82 ± 0.06^e^
SPI-6	22.61 ± 0.57^f^	30.12 ± 1.21^f^	51.31 ± 0.85^e^	71.38 ± 1.55^f^	83.52 ± 0.49^f^	7.51 ± 0.64^bcd^	4.10 ± 0.04^e^	3.49 ± 0.15^d^	2.90 ± 0.06^e^

Values followed by the different letter in the same column are significantly different at the 5% level.

### 3.4 Gel stress relaxation

The stress relaxation curves of rice noodle gels containing exogenous proteins are presented in Figure 1B, and the corresponding fitting parameters obtained from the three-element Maxwell model are summarized in Table 4. Following WG addition, the values of *E*_0_, *E*_1_, *η,* and *τ* increased significantly with increasing protein content. The WG-6 sample exhibited the highest values, with *E*_0_, *E*_1_, and *η* reaching 4.14 MPa, 10.16 MPa, and 29.53 MPa·s, respectively. Meanwhile, the relaxation time increased to 2.91 s. These results indicate that WG substantially enhanced the elastic and viscoelastic properties of the gel network, resulting in greater resistance to deformation and a slower stress relaxation process (25). The strengthening effect became increasingly pronounced as the WG content increased. In contrast, SPI produced only a slight increase in stress relaxation parameters at low addition levels. When the SPI content reached 6%, all fitted parameters showed a declining trend. Overall, WG was considerably more effective than SPI in reinforcing the elastic network structure of rice noodle gels and improving their deformation resistance. The hydrophobic polypeptide chains of WG can interact closely with starch molecules through hydrophobic interactions and become incorporated into the starch gel matrix, thereby forming a more stable composite network structure and enhancing the elasticity and viscosity of the gel (26). In contrast, SPI exhibits strong hydrophilic properties and, at high addition levels, competes extensively with starch for available water. This competition thereby reduces the integrity and strength of the starch gel network and ultimately leads to a decrease in gel elasticity.

**Table 4:** Stress relaxation of rice flour gels with exogenous protein addition.

Sample	*E*_0_/MPa	*E*_1_/MPa	*η*/MPa·s	*τ*/s
Control	3.15 ± 0.08^c^	5.20 ± 0.04^e^	8.75 ± 0.15^e^	1.68 ± 0.04^d^
WG-1	3.53 ± 0.23^b^	7.63 ± 0.20^c^	14.55 ± 0.98^d^	1.91 ± 0.14^d^
WG-3	3.61 ± 0.12^b^	9.14 ± 0.55^b^	21.87 ± 2.72^b^	2.39 ± 0.19^bc^
WG-6	4.14 ± 0.20^a^	10.16 ± 0.46^a^	29.53 ± 1.60^a^	2.91 ± 0.17^a^
SPI-1	3.18 ± 0.19^c^	6.43 ± 0.39^d^	11.06 ± 0.59^e^	1.72 ± 0.04^d^
SPI-3	3.48 ± 0.17^b^	7.79 ± 0.53^c^	17.20 ± 0.56^c^	2.21 ± 0.08^c^
SPI-6	2.78 ± 0.13^d^	6.23 ± 0.09^d^	16.23 ± 0.93^cd^	2.60 ± 0.11^b^

Values followed by the different letter in the same column are significantly different at the 5% level.

### 3.5 Low-field nuclear magnetic resonance

**Figure 2:**
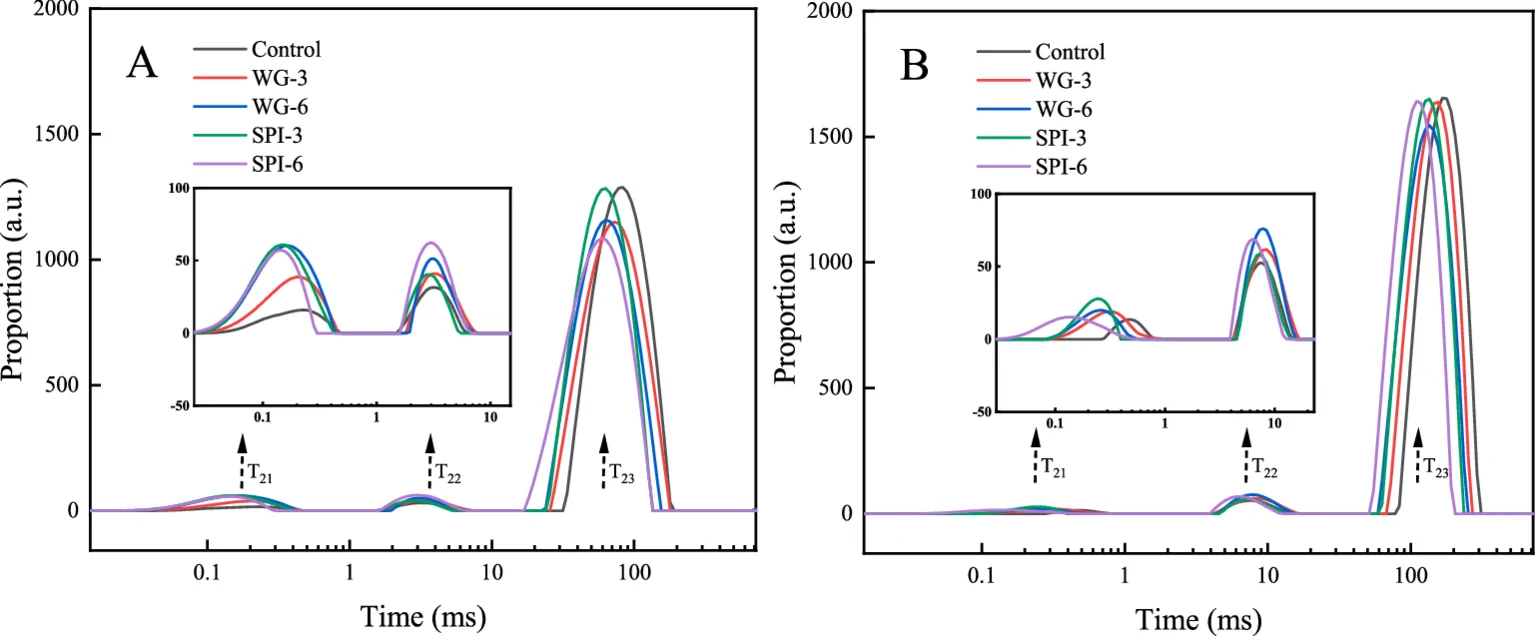
Effect of exogenous protein on transverse relaxation time and peak intensity of rice flour gel aged for 1 d **(A)** and 7 d **(B)**. Control: pure rice flour gels; WG-3 and WG-6: rice flour gels with 3 and 6% wheat gluten addition, respectively; SPI-3 and SPI-6: rice flour gels with 3 and 6% soy protein isolate addition, respectively.

Water migration and redistribution within the gel matrix are among the most important characteristics associated with starch retrogradation. The transverse relaxation time (*T*₂) obtained by LF-NMR is a sensitive indicator of water mobility and can effectively reflect changes in the binding state of water molecules within starch gel systems. All spectra present three separate peaks assigned to three water fractions with different mobilities: tightly bound water (*T*₂₁) tightly combined with solid matrix and showing the lowest fluidity, weakly bound water (*T*₂₂) with moderate mobility, and highly mobile free water (*T*₂₃). Their corresponding relative peak areas are defined as *A*₂₁, *A*₂₂, and *A*₂₃, respectively (27). As shown in Figure 2 and Table 5, the overall *T*₂ distribution shifted toward longer relaxation times as the storage period increased from 1 to 7 days. This result indicates an increase in water mobility and a reduction in water binding strength within the gel matrix during storage. This is reflected macroscopically by increased hardness and a greater tendency for water separation (28).

**Table 5:** Transverse relaxation time and peak intensity of rice flour gels with exogenous protein addition.

Time	Sample	*T*_21_/ms	*T*_22_/ms	*T*_23_/ms	*A*_21_/a.u.	*A*_22_/a.u.	*A*_23_/a.u.
1 d	Control	0.20 ± 0.01^a^	6.83 ± 0.18^a^	102.34 ± 1.77^a^	16.04 ± 0.08^e^	31.58 ± 0.11^e^	1288.27 ± 9.15^a^
	WG-3	0.18 ± 0.01^a^	6.37 ± 0.47^a^	95.48 ± 2.62^b^	38.68 ± 0.13^d^	41.08 ± 0.08^c^	1150.53 ± 8.43^b^
	WG-6	0.17 ± 0.03^a^	5.17 ± 0.25^b^	89.07 ± 1.34^c^	60.46 ± 0.07^b^	51.43 ± 0.17^b^	1157.84 ± 4.09^b^
	SPI-3	0.16 ± 0.03^a^	5.17 ± 0.35^b^	89.53 ± 1.17^c^	60.89 ± 0.04^a^	40.41 ± 0.35^d^	1283.83 ± 5.63^a^
	SPI-6	0.16 ± 0.01^a^	4.50 ± 0.16^b^	83.10 ± 2.52^d^	57.23 ± 0.03^c^	62.37 ± 0.18^a^	1084.53 ± 12.13^c^
7 d	Control	0.30 ± 0.03^a^	7.84 ± 0.16^a^	126.04 ± 1.74^a^	13.63 ± 0.07^e^	52.57 ± 0.07^e^	1654.52 ± 13.17^a^
	WG-3	0.28 ± 0.01^a^	7.32 ± 0.20^b^	122.17 ± 2.02^ab^	19.13 ± 0.06^c^	61.71 ± 0.04^c^	1637.44 ± 7.95^a^
	WG-6	0.26 ± 0.01^ab^	7.24 ± 0.11^b^	117.59 ± 4.02^b^	19.98 ± 0.13^b^	76.09 ± 0.08^a^	1545.03 ± 14.89^b^
	SPI-3	0.28 ± 0.01^a^	6.83 ± 0.04^c^	117.33 ± 2.06^b^	27.94 ± 0.25^a^	58.62 ± 0.07^d^	1650.29 ± 5.22^a^
	SPI-6	0.23 ± 0.01^b^	5.17 ± 0.21^d^	102.34 ± 3.37^c^	15.29 ± 0.28^d^	68.97 ± 0.04^b^	1641.6 ± 8.95^a^

Values followed by the different letter in the same column are significantly different at the 5% level.

In the protein-enriched systems, the transverse relaxation time *T*_2_ shifted to the left compared with the control group. This phenomenon can be attributed to competition between proteins and starch for water molecules, which increases the proportion of strongly bound water and weakly bound water closely associated with biological macromolecules. Accordingly, the corresponding peak signal intensities *A*_21_ and *A*_22_ increased. This result indicates that the protein addition effectively enhances the water-holding capacity of rice noodle gel matrix, thereby retarding water migration and redistribution during starch retrogradation. Consequently, the increase in gel hardness during storage was attenuated, providing direct evidence of the inhibitory effect of proteins on starch retrogradation. At the same storage period, the rightward shift of *T*₂ in the SPI group was less pronounced than that observed in the WG group at the same supplementation level, indicating that SPI was more effective in suppressing water-phase transformation and exhibited the strongest inhibitory effect on starch retrogradation.

### 3.6 FTIR

**Figure 3:**
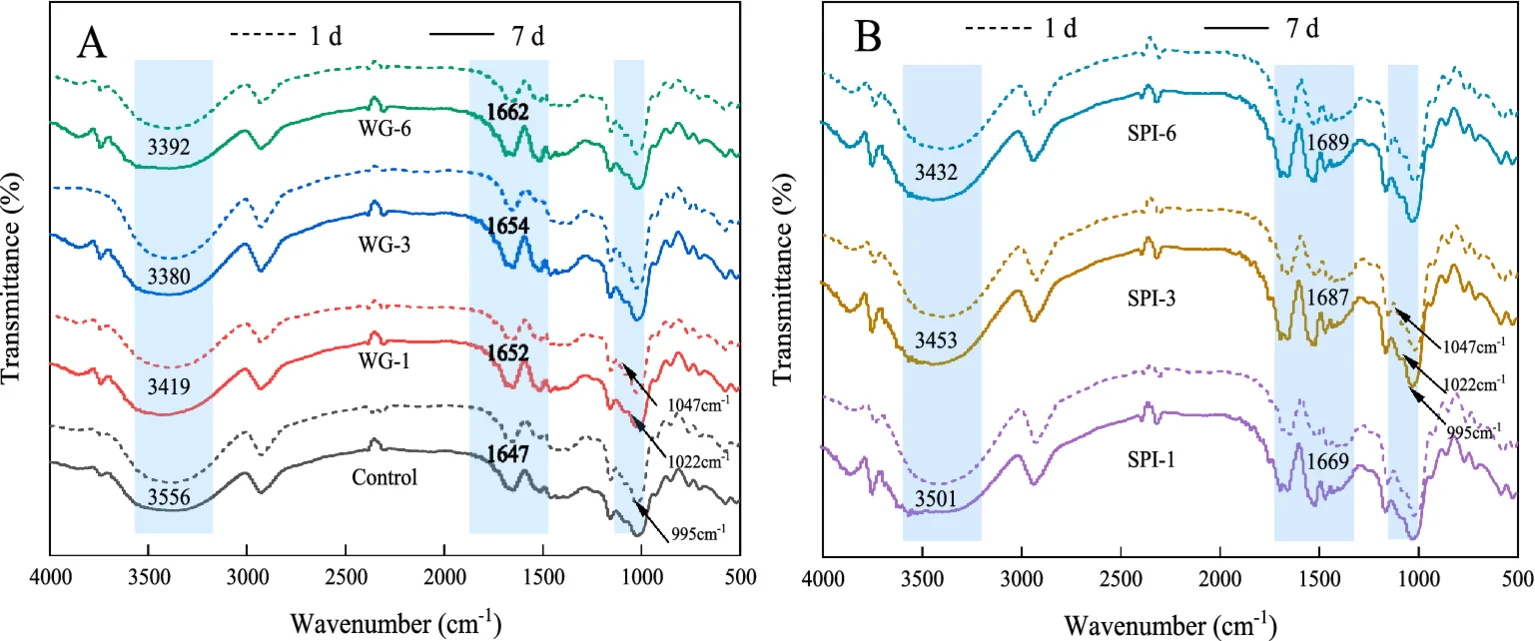
Fourier transform infrared spectra of rice flour gel with added wheat gluten **(A)** and soy protein isolate **(B)** during different aging times. Control: pure rice flour gels; WG-1, WG-3, and WG-6: rice flour gels with 3 and 6% wheat gluten addition, respectively; SPI-1, SPI-3, and SPI-6: rice flour gels with 3 and 6% soy protein isolate addition, respectively.

The FTIR spectra of rice flour gels containing exogenous proteins after 1 d and 7 d of storage are shown in Figure 3. No new functional groups or noticeable shifts in characteristic absorption peaks were observed in any of the starch–protein gel systems, indicating that the interaction between starch and protein mainly occurs through non-covalent interactions, particularly hydrogen bonding. Hydrogen bonds, as the primary driving force of starch retrogradation, are reflected by changes in the broad absorption band within the range of 3,000–3,600 cm^−1^. An absorption band centered at approximately 3,400 cm^−1^, corresponding to O–H stretching vibrations, was observed in all samples. With increasing storage time, the absorption band gradually widened and shifted toward lower wavenumbers following protein addition, indicating an enhancement in hydrogen-bond interactions within the gel network. The absorption band near 1650 cm^−1^ is associated with the O-H stretching vibration of bound water. Compared with the pure starch gel, protein incorporation significantly increased the intensity of this band, indicating an enhanced water-holding capacity. This effect is attributed to alterations in the binding state of water molecules induced by protein addition, which is consistent with the findings reported by Luo et al. (29).

The absorbance at 1047 cm^−1^, 1022 cm^−1^, and 995 cm-1 is associated with the crystalline regions, amorphous regions, and helical structure of starch (30). As shown in Table 6, the addition of proteins at different concentrations reduced the degree of structural order in the gel system. This phenomenon may be related to the gelation behavior of proteins, which compete with starch for available water and thereby limit starch molecular reassociation during storage, suppressing the formation of long-range ordered crystalline structures. Furthermore, hydrophobic interactions between proteins and starch weaken the interaction between starch molecules and restrict the formation of inclusion complexes. As a result, the molecular rearrangement and recrystallization processes associated with starch retrogradation are effectively retarded (31).

**Table 6:** Short-range order of rice flour gels with exogenous protein addition.

Sample	1 d		7 d	
	*R*₁₀₄₇/₁₀₂₂	*R*₉₉₅/₁₀₂₂	*R*₁₀₄₇/₁₀₂₂	*R*₉₉₅/₁₀₂₂
Control	0.98 ± 0.03^a^	3.29 ± 0.06^a^	1.21 ± 0.04^a^	3.54 ± 0.05^a^
WG-1	0.97 ± 0.08^a^	3.21 ± 0.21^a^	1.13 ± 0.03^b^	3.34 ± 0.04^b^
WG-3	0.95 ± 0.05^a^	2.67 ± 0.07^b^	1.03 ± 0.05^c^	3.33 ± 0.04^b^
WG-6	0.94 ± 0.10^a^	2.63 ± 0.20^b^	1.02 ± 0.04^c^	3.11 ± 0.01^c^
SPI-1	0.97 ± 0.06^a^	3.20 ± 0.15^a^	1.14 ± 0.02^b^	3.30 ± 0.11^b^
SPI-3	0.95 ± 0.05^a^	2.77 ± 0.15^b^	1.01 ± 0.02^c^	2.86 ± 0.06^d^
SPI-6	0.78 ± 0.05^b^	2.69 ± 0.06^b^	0.85 ± 0.04^d^	2.84 ± 0.03^d^

Values followed by the different letter in the same column are significantly different at the 5% level.

### 3.7 CLSM

**Figure 4:**
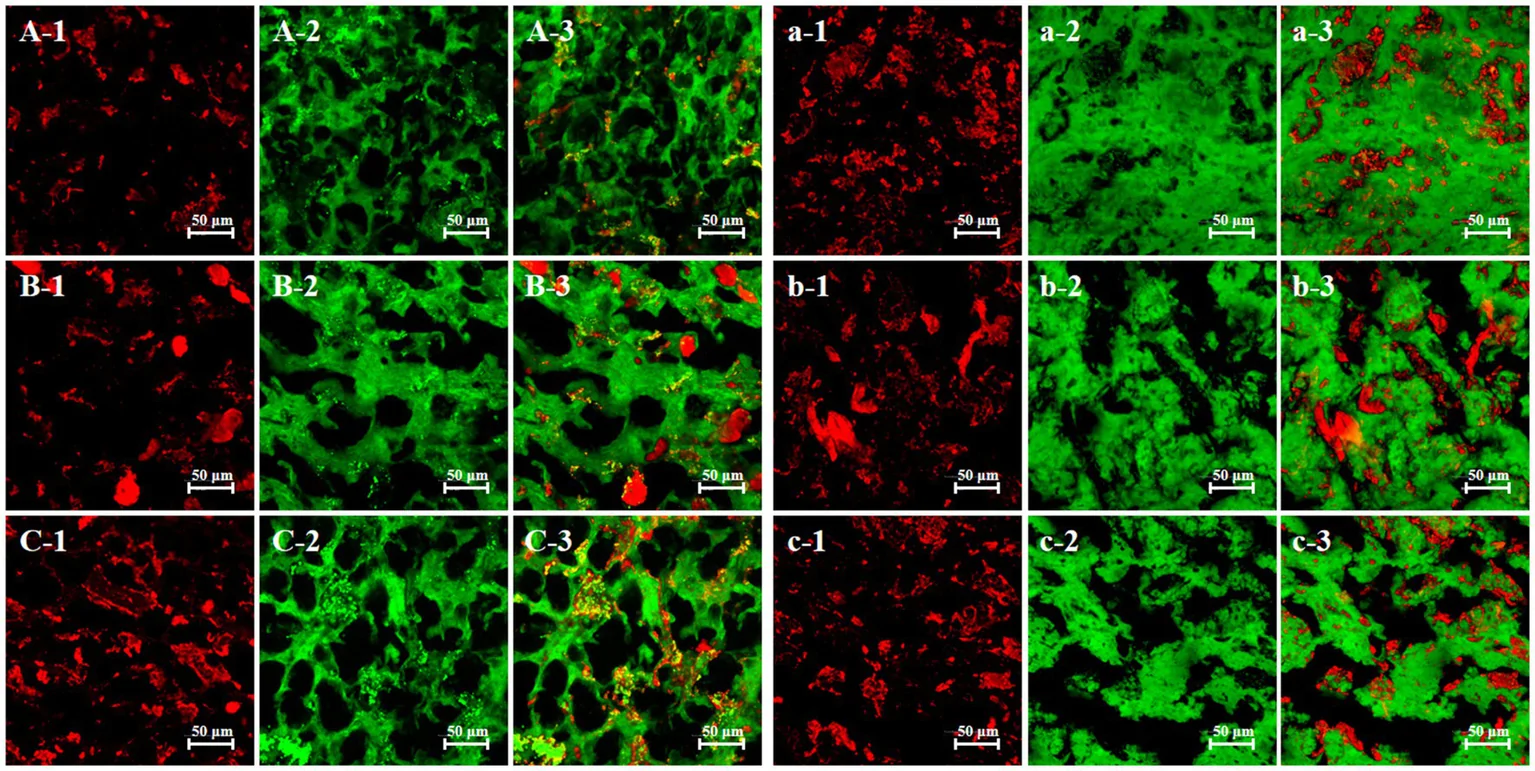
CLSM observations of gel microstructure affected by exogenous proteins. Images **(A1–C3)** correspond to the fresh control, WG-6 and SPI-6 samples, while **(a1–c3)** represent the respective samples after 7 days of aging. Panels 1, 2 and 3 display the morphologies of protein network, starch network and protein-starch composite network in sequence. Control: pure rice flour gels; WG-6: rice flour gels with 6% wheat gluten addition; SPI-6: rice flour gels with 6% soy protein isolate addition.

To further clarify the distribution of starch and protein within the rice noodle gel, samples were examined using CLSM. The CLSM images exhibited distinct fluorescence regions, with starch stained green by FITC and proteins stained red by rhodamine B (Figures 4A-1, B-1, C-1, a-1, b-1, c-1) (32). As shown in Figures 4A-2, B-2, C-2, the green fluorescence formed a continuous three-dimensional network, indicating that the starch granules had completely gelatinized, ruptured, and fused into an integrated gel structure (33). The red fluorescence images in Figures 4A-3, B-3, C-3 revealed that proteins were distributed both on the surface of the starch gel skeleton and within the pores and voids of the starch network (34). Compared with the control (Figure 4A-3) group, WG-6 (Figure 4B-3) and SPI-6 (Figure 4C-3) exhibited noticeably larger red fluorescence regions. Quantitative image analysis (Table 7) further confirmed that the protein area fraction increased with protein addition, while no significant difference was observed between WG-6 and SPI-6. In addition, it can be seen from Figure 4B-3 that after adding WG, proteins aggregate into discrete particles dispersed throughout the starch matrix. This phenomenon is likely related to the poor water solubility of WG, which limits its homogeneous distribution within the gel network and may partially explain its weaker inhibitory effect on starch retrogradation compared with SPI.

**Table 7:** Effects of exogenous proteins on the protein area fraction of rice flour gels.

Time	Sample	Protein area/×10^3^μm^2^	Protein percentage area/%
0 d	Control	145.19 ± 7.38^b^	13.85 ± 0.70^c^
	WG-6	209.96 ± 1.66^a^	20.35 ± 0.35^b^
	SPI-6	217.32 ± 2.04^a^	23.12 ± 0.31^a^
7 d	Control	175.48 ± 3.78^b^	20.50 ± 0.51^b^
	WG-6	286.17 ± 8.96^a^	27.30 ± 0.85^a^
	SPI-6	277.15 ± 4.09^a^	26.70 ± 0.01^a^

Values followed by the different letter in the same column are significantly different at the 5% level.

After 7 days of retrogradation, all samples exhibited signs of starch retrogradation (Figures 4a-2, b-2, c-2), characterized by a more continuous and compact network with fewer and smaller pores corresponding to the increased hardness observed at the macroscopic level (35). These structural changes were most pronounced in the control group (Figure 4a-2). In contrast, the SPI-6 sample (Figure 4c-2) displayed the slowest degree of network rearrangement, retained a relatively loose microstructure, and exhibited the least structural densification during storage, indicating a stronger ability to retard starch retrogradation than WG. In addition, the protein fluorescence signal became brighter and more concentrated after retrogradation (Figures 4a-3, b-3, c-3). This observation may be attributed to enhanced interactions and cross-linking between starch and protein molecules during storage (36), resulting in an increased relative protein signal. These findings are consistent with the results reported by Dai et al. (16).

### 3.8 Supramolecular structure

**Figure 5:**
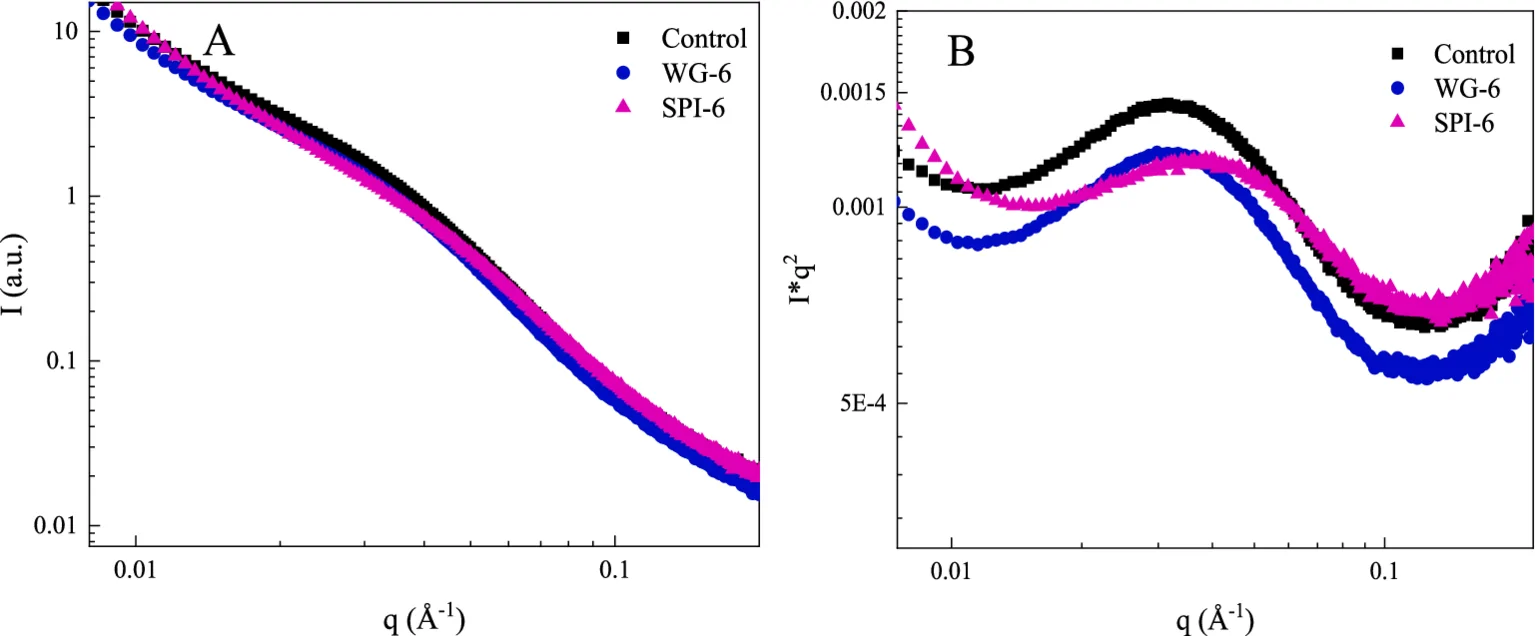
SAXS patterns **(A)** and Lorentz-corrected scattering patterns **(B)** of rice noodles incorporated with WG-6 and SPI-6. Control: pure rice noodles; WG-6: rice noodles with 6% wheat gluten addition; SPI-6: rice noodles with 6% soy protein isolate addition.

SAXS can effectively investigate the lamellar structure of rice noodles through the electron cloud density contrast between crystalline and amorphous regions of starch. The original SAXS profiles (Figure 5A) and the corresponding Lorentz -corrected scattering curves are shown in Figure 5B. The control, WG-6, and SPI-6 groups show characteristic scattering peaks at *q* values of 0.32 nm^−1^, 0.36 nm^−1^, and 0.38 nm^−1^, respectively. According to the Bragg equation, the corresponding semi-crystalline lamellar thickness *d*_Bragg_ were calculated to be 19.64 nm, 17.55 nm, and 16.47 nm. These results suggest that protein incorporation restricts the structural reorganization of starch during retrogradation, leading to the formation of thinner lamellar structures, with SPI exhibiting a stronger effect than WG.

The normalized one-dimensional correlation function obtained by Fourier transformation is summarized in Table 8. For all samples, the thickness *d*_c_ of the crystalline layer was smaller than that of the amorphous layer *d*_a_. This phenomenon may be attributed to the disruption of amylopectin double helices during gelatinization, whereas only a limited number of amylose molecules participate in double-helix formation during the early stages of retrogradation. Consequently, a large number of amylopectin remains in a disordered state, resulting in relatively loose newly formed lamellar structures. After protein addition, *d*_c_ and *d*_a_ decreased significantly. In particular, the SPI-6 sample exhibited *d*_c_ and *d*_a_ values of 2.35 nm and 11.40 nm, respectively, which were significantly lower than those of the WG-6 and control group. This result suggests that proteins interact with starch molecules through hydrogen bonding and other non-covalent interactions, thereby restricting starch chain migration and rearrangement within the amorphous regions. As a result, the growth of crystalline domains is suppressed, and starch retrogradation is delayed. The calculated fractal parameter *α* ranged from 1 to 3 for all samples, indicating that the gel system exhibited typical mass-fractal characteristics. Protein addition significantly increased the *α* value, reaching 2.37 and 2.48 for the WG-6 and SPI-6 groups, respectively, both of which were significantly higher than that of the control group. This indicates that the addition of proteins forms a denser and more complex internal network structure, which is related to the enhanced interactions between starch and protein molecules, and further supports its inhibitory effect on starch retrogradation.

**Table 8:** Lamellar structure parameters of rice noodles incorporated with WG-6 and SPI-6.

Sample	*q*/nm^−1^	*d*_Bragg_ /nm	*d*/nm	*d*_c_/nm	*d*_a_/nm	*α*
Control	0.32 ± 0.01^c^	19.64 ± 0.35^a^	16.22 ± 0.09^a^	2.71 ± 0.02^a^	13.51 ± 0.07^a^	2.15 ± 0.04^b^
WG-6	0.36 ± 0.01^b^	17.55 ± 0.28^b^	15.10 ± 0.09^b^	2.54 ± 0.04^b^	12.56 ± 0.12^b^	2.37 ± 0.04^a^
SPI-6	0.38 ± 0.01^a^	16.47 ± 0.27^c^	13.75 ± 0.14^c^	2.35 ± 0.04^c^	11.40 ± 0.10^c^	2.48 ± 0.04^a^

Values followed by the different letter in the same column are significantly different at the 5% level.

### 3.9 Quality determination of rice noodles

#### 3.9.1 Cooking characteristics and color of rice noodles

Table 9 summarizes the effects of exogenous protein addition on the color attributes and cooking characteristics of rice noodles. Among the color parameters, the yellowness value exhibited the most pronounced changes and was closely associated with the reduction in whiteness. The control group has the lowest b* value and the highest whiteness. With increasing levels of WG and SPI, the b* values of both groups increased significantly, reaching 12.43 and 12.80 in the WG-6 and SPI-6 groups, respectively. Correspondingly, the whiteness values decreased to 63.71 and 65.54. These results indicate that the addition of proteins enhanced the yellowness and reduced the whiteness of rice noodles (37). The observed color changes may be caused by the intrinsic composition of the added proteins and their participation in browning reactions during processing and storage (38): WG contains relatively high levels of sulfur-containing amino acids and phenolic compounds, which may promote pigment formation through Maillard and oxidation reactions. Similarly, the existence of polyphenol oxidase, flavonoids, and unsaturated fatty acids in SPI may contribute to both enzymatic and non-enzymatic browning processes, resulting in increased yellowness.

**Table 9:** Whiteness and cooking characteristics of rice noodles with exogenous protein addition.

Sample	*L* ^*^	*a* ^*^	*b* ^*^	*W*	Cooking loss/%
Control	67.26 ± 0.03^c^	−0.92 ± 0.07^c^	8.20 ± 0.02^f^	66.23 ± 0.03^a^	7.37 ± 0.88^a^
WG-1	66.95 ± 0.20^c^	−1.04 ± 0.05^d^	9.35 ± 0.09^e^	65.63 ± 0.21^b^	4.10 ± 0.30^c^
WG-3	67.31 ± 0.05^bc^	−1.07 ± 0.07^d^	11.08 ± 0.15^c^	65.47 ± 0.05^b^	3.61 ± 0.20^c^
WG-6	65.91 ± 0.27^e^	−0.72 ± 0.06^b^	12.43 ± 0.12^b^	63.71 ± 0.29^d^	3.30 ± 0.24^c^
SPI-1	66.50 ± 0.19^d^	−0.89 ± 0.07^c^	9.95 ± 0.19^d^	65.04 ± 0.24^c^	6.67 ± 0.28^b^
SPI-3	67.68 ± 0.04^ab^	−0.88 ± 0.08^c^	11.22 ± 0.27^c^	65.77 ± 0.05^b^	5.77 ± 1.05^b^
SPI-6	68.01 ± 0.41^a^	−0.60 ± 0.06^a^	12.80 ± 0.19^a^	65.54 ± 0.32^b^	6.86 ± 0.33^ab^

Values followed by the different letter in the same column are significantly different at the 5% level.

As the levels of WG and SPI increased, the cooking loss of rice noodles decreased significantly, with a more pronounced reduction observed in the WG-treated samples. Among them, the WG-6 group exhibited the lowest cooking loss of only 3.30%. This result may be attributed to the ability of WG to interact with starch molecules through hydrophobic interactions, forming a more stable and continuous starch–protein gel network. Such a network effectively limits the leaching of starch, soluble proteins, and other components during cooking (39), thus reducing cooking loss.

#### 3.9.2 Textural characteristics of cooked rice noodles

As shown in Table 10, the addition of WG and SPI significantly affected the textural properties of rice noodles, although these effects depended on protein type and supplementation level. Compared with the control group, WG generally improved the tensile properties of rice noodles, suggesting enhanced ductility and structural integrity. When the WG content reached 6%, the breaking force decreased, implying that excessive addition may partially disrupt the continuity of the gel network. Regarding hardness, WG supplementation significantly increased the hardness of rice noodles after boiling, and the hardness values generally increased with increasing WG content. In contrast, the addition of SPI significantly reduced the breaking force and breaking distance of rice noodles, and these parameters decreased progressively with increasing SPI content. This finding suggests that SPI may interfere with the formation of a continuous starch gel network, thereby reducing toughness and extensibility. A similar trend was observed for hardness. Although the SPI-1 group exhibited a slight increase in initial hardness (225.39 g), the hardness values of SPI-3 and SPI-6 decreased significantly. In particular, the hardness of the SPI-6 group was significantly lower than those of the other groups. This result indicates that high levels of SPI markedly soften the rice noodle structure (40), which is consistent with the gel hardness and stress-relaxation analyses presented above. The observed softening effect may be associated with the ability of SPI to inhibit starch molecular reassociation and retrogradation, thereby limiting the formation of a rigid gel network. Similar findings have been reported by Yang et al. (9), who showed that SPI incorporation significantly reduced the hardness of starch gels while simultaneously suppressing starch retrogradation.

**Table 10:** Textural properties of rice noodles with exogenous protein addition.

Sample	Adhesiveness/g·sec	Hardness/g	Hardness-7/g	ΔHardness-7/g·d^−1^	Hardness-14 /g	ΔHardness-14/g·d^−1^	Tensile strength/g	Resistance/mm
Control	40.32 ± 0.81^a^	185.29 ± 0.45^c^	330.16 ± 0.85^a^	20.70 ± 0.19^a^	397.72 ± 1.10^a^	15.18 ± 0.05^a^	25.50 ± 0.45^a^	52.40 ± 0.36^b^
WG-1	31.58 ± 0.16^cd^	213.58 ± 0.70^b^	324.97 ± 0.59^b^	15.91 ± 0.01^b^	376.35 ± 2.83^b^	11.63 ± 0.15^b^	25.86 ± 1.14^a^	53.07 ± 1.32^b^
WG-3	30.98 ± 0.80^d^	226.38 ± 7.40^a^	256.23 ± 1.10^d^	4.27 ± 0.90^d^	276.42 ± 0.72^d^	3.58 ± 0.47^d^	26.06 ± 0.25^a^	63.05 ± 3.06^a^
WG-6	32.34 ± 0.07^cd^	224.29 ± 1.85^a^	239.92 ± 1.24^e^	2.23 ± 0.08^e^	254.02 ± 1.90^e^	2.13 ± 0.01^e^	23.37 ± 0.35^b^	51.97 ± 0.01^b^
SPI-1	35.86 ± 1.05^b^	225.39 ± 2.00^a^	262.18 ± 0.21^c^	5.26 ± 0.32^c^	289.24 ± 5.25^c^	4.57 ± 0.23^c^	20.93 ± 0.10^c^	42.99 ± 1.09^c^
SPI-3	33.67 ± 0.39^bc^	175.96 ± 0.40^d^	186.03 ± 1.73^f^	1.44 ± 0.30^e^	197.82 ± 1.61^f^	1.56 ± 0.08^e^	17.54 ± 1.00^d^	33.13 ± 2.36^d^
SPI-6	30.79 ± 1.95^d^	105.47 ± 4.67^e^	115.52 ± 4.60^g^	1.44 ± 0.01^e^	127.20 ± 1.16^g^	1.55 ± 0.42^e^	14.18 ± 1.15^e^	27.48 ± 1.51^e^

Values followed by the different letter in the same column are significantly different at the 5% level. Hardness-7 and Hardness-14 refer to the hardness of rice noodles after 7 and 14 days of retrogradation, respectively. ΔHardness-7 and ΔHardness-14 represent the hardness change rates over 7-day and 14-day retrogradation periods.

#### 3.9.3 Hardness changes of cooked rice noodles

As shown in Table 10, the hardness of all samples increased with storage time, reflecting the continued progression of starch retrogradation. However, the addition of proteins significantly influenced both the retrogradation process and the rate of hardness development in rice noodles. The control group exhibited the highest hardness after 7 d and 14 d of retrogradation, as well as the largest ΔHardness value, indicating the greatest extent of retrogradation-induced hardening. Increasing the WG content from 1 to 6% reduced the hardness at 7 d from 324.97 g to 239.92 g, and markedly decreased ΔHardness from 15.91 to 2.23 g·d^−1^. Similarly, increasing the SPI addition content from 1 to 6% reduced the hardness from 262.18 g to 115.52 g, and decreased ΔHardness from 5.26 to 1.44 g·d^−1^. This indicates that both WG and SPI effectively suppressed hardness development during storage, with the inhibitory effect becoming more pronounced as the protein addition level increased. However, at equivalent supplementation levels, SPI generally produced a greater reduction in both hardness and ΔHardness than WG, particularly at higher addition levels. This finding suggests that SPI is more effective in suppressing starch molecular reassociation and the associated hardening process during storage. The superior performance of SPI is consistent with the results obtained from the analyses of water distribution and gel texture. Compared with WG, SPI exhibited a stronger ability to improve water holding capacity and retard water migration and redistribution during storage, thereby slowing the increase in gel hardness. Although the SPI-6 group significantly slowed the increase rate of hardness, texture profile analysis revealed that tensile strength and breaking distance of rice noodles were greatly reduced simultaneously. For fresh wet rice noodles, reduced hardness induced by retrogradation does not equal superior quality. Excessive softening, deteriorated extensibility and insufficient supporting capacity of the network structure are also regarded as quality defects; thus, excessive SPI addition is not recommended in practical applications.

Notably, the amylose content of the rice raw material used in this study is slightly below the optimal range reported for high-quality indica rice noodle processing. Given that amylose content is a core factor governing starch retrogradation kinetics and gel textural properties, the modification efficacy of exogenous proteins may differ across rice matrices with varying amylose levels. We speculate that the performance difference between WG and SPI would be more pronounced in higher-amylose rice systems. This hypothesis remains to be systematically verified in future studies to clarify the applicability of exogenous protein modification strategies across diverse rice raw materials.

## 4 Conclusion

In this study, the effects of WG and SPI at supplementation levels of 0, 1, 3 and 6% on the retrogradation behavior of rice starch gels and the quality characteristics of fresh wet rice noodles were systematically investigated. Both types of exogenous proteins can effectively reduce the paste viscosity and setback values, enhance gelatinization temperature and gel water-holding capacity, suppress starch recrystallization, and retarded retrogradation-induced hardening. In general, these effects became more pronounced with increasing protein addition levels. Between the two proteins, SPI possesses a superior capacity to slow starch retrogradation through more efficient inhibition of water migration. Notably, supplementation with 3% SPI delivers a favorable balance between anti-retrogradation performance and sensory attributes. Nevertheless, high SPI dosages soften the noodle matrix and significantly impair tensile characteristics. In comparison, WG at addition levels of 3–6% reinforces the gel network more prominently, which enhances the textural and tensile properties of rice noodles and achieves a more substantial reduction in cooking loss.

## Data Availability

The original contributions presented in the study are included in the article/supplementary material, further inquiries can be directed to the corresponding authors.
